# Emergent *d*‑Wave Altermagnetism in Orthogonally Twisted Bilayer CrPS_4_

**DOI:** 10.1021/acsmaterialsau.6c00086

**Published:** 2026-06-17

**Authors:** Alberto M. Ruiz, Diego López-Alcalá, Rafael González-Hernández, José J. Baldoví

**Affiliations:** † Instituto de Ciencia Molecular, 16781 Universitat de València, Catedrático José Beltrán 2, 46980 Paterna, Spain; ‡ Departamento de Física y Geociencias, 28003 Universidad del Norte, Km. 5 Vía Antigua Puerto Colombia, 081007 Barranquilla, Colombia

**Keywords:** altermagnetism, twistronics, spintronics, CrPS_4_, first-principles calculations

## Abstract

Twistronics is a powerful strategy to engineer novel quantum states by controlling the relative orientation between layered materials. Here, we demonstrate that an orthogonally twisted bilayer CrPS_4_ shows *d*-wave altermagnetism driven purely by structural rotation. Symmetry analysis reveals that the 90° twisted stacking breaks the combined inversion and time-reversal symmetry, leading to an in-plane 2-fold rotation operation connecting opposite spin sublattices, enabling altermagnetism. First-principles calculations demonstrate a sizable nonrelativistic spin splitting of up to 68 meV around the Fermi level. Additionally, we show that the altermagnetic state can be further stabilized through interlayer compression and modulation of the on-site Coulomb interaction. The resulting band structure exhibits pronounced spin-dependent anisotropy, enabling efficient spin to charge conversion reaching ∼50% near the Fermi level and sizable giant magnetoresistance. These results establish twisted CrPS_4_ as a realistic platform for altermagnetism and highlight twistronics as a versatile route for advanced spintronics applications.

## Graphical abstract

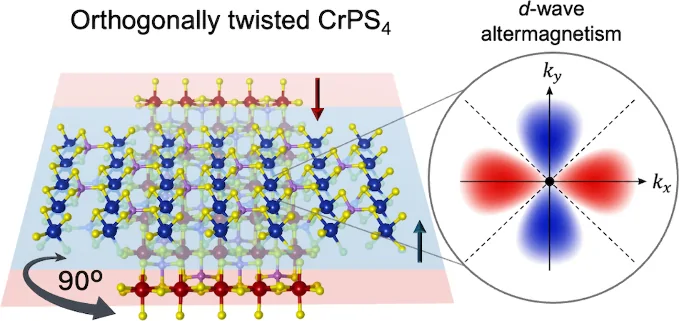

Twistronics—the controlled rotational alignment of two-dimensional (2D) materials—has emerged as a powerful route for engineering novel quantum states, leading to phenomena such as moiré flat bands, unconventional superconductivity, or ferroelectricity.^1–7^ In magnetic van der Waals (vdW) materials, twisting allows the modification of the symmetry relations between spin sublattices without chemical substitution, influencing their magnetic response. For instance, in CrI_3_, small twist angles induce an interplay between monoclinic and rhombohedral stacking configurations associated with interlayer antiferromagnetic (AF) and ferromagnetic (FM) states, respectively.^8–11^ The resulting competition between stacking-dependent exchange and magnetic anisotropy generates noncollinear topological spin textures and theoretically predicted spin–orbit coupling (SOC)-driven local ferroelectric polarization.^12–16^

Within this broader magnetic landscape, altermagnetism has recently emerged as a magnetic state characterized by a momentum-dependent spin splitting in the absence of net magnetization.^17–20^ Therefore, altermagnets combine key advantages of both FM and AF materials such as absence of stray fields, robustness against external perturbations, and ultrafast spin dynamics, while enabling sizable spin-polarized electronic responses.^21–23^ In altermagnets, spin splitting originates from crystal symmetry rather than SOC and exhibits characteristic patterns in momentum space. Those can be classified as *d*-, *g*-, or *i*-wave type corresponding to 2, 4, or 6 spin degenerate nodal surfaces crossing the Γ point, respectively.^24,25^

Twisting layered antiferromagnets, besides other stacking-driven approaches,^26–28^ has recently been proposed as an effective pathway for altermagnetism in 2D systems.^29–32^ Most reported candidates to date rely on relatively small twisting angles between layers, resulting in higher-order *i*-wave spin splitting patterns. Consequently, their symmetry enforces multiple sign changes of the spin polarization, often leading to equivalent group velocities for opposite spin channels and suppressing spin-polarized currents. In contrast, *d*-wave altermagnets are particularly desirable, as their reduced number of spin-degenerate nodal surfaces results in anisotropic group velocities that enable spin-polarized transport.^33–36^ In this context, a 90° rotation between adjacent layers provides a natural platform to realize *d*-wave altermagnetism by generating two orthogonal spin channels with opposite spin polarization.^29,37^

Recently, 90° twisted bilayer CrSBr has been theoretically proposed as a *d*-wave altermagnet based on the AF interlayer coupling of the pristine system and the experimental realization of the orthogonally stacked architecture.^29,38,39^ However, its in-plane anisotropy results in two decoupled layers in the rotated form, where spins are independently aligned along their respective easy axes.^39,40^ This behavior, combined with its extremely weak interlayer antiferromagnetism in the pristine phase, precludes altermagnetism.^41–43^ In contrast, CrPS_4_ emerges as a more robust and promising platform. CrPS_4_ is a vdW A-type AF semiconductor and, importantly, exhibits out-of-plane magnetic anisotropy together with significantly stronger interlayer AF coupling.^44–46^ These characteristics suggest that interlayer antiferromagnetism may be preserved under rotational stacking, providing an ideal platform for altermagnetism.

In this work, we demonstrate that orthogonally twisted bilayer CrPS_4_ is a *d*-wave altermagnet by combining first-principles calculations and symmetry analysis. In particular, we show that a 90° rotation between adjacent layers leads to opposite spin sublattices connected through an in-plane 2-fold rotation operation rather than spatial inversion. This configuration stabilizes a *d*-wave altermagnetic (AM) state, giving rise to a momentum-dependent spin splitting around the Fermi level of 68 meV even in the absence of SOC. Moreover, the twisted structure enables efficient spin to charge conversion near the Fermi level, reaching values approaching 50%. Our results establish twisted CrPS_4_ as an experimentally accessible candidate for altermagnetism.

An illustration of the chemical structure of bilayer CrPS_4_ is shown in Figure 1a. Each layer consists of edge-sharing CrS_6_ octahedra and PS_4_ tetrahedra extended along the *b* and *a* directions, respectively, resulting in pronounced in-plane structural anisotropy.^47,48^ To characterize the electronic and magnetic properties of CrPS_4_, we perform first-principles calculations within the DFT+*U* framework. The optimized lattice parameters are *a* = 10.88 Å and *b* = 7.28 Å, showing an interlayer separation of 2.52 Å.^47^ The interlayer exchange coupling (*J*_int_) is evaluated from the energy difference between AF and FM configurations. Our results yield *J*_int_ = −0.59 meV/Cr, correctly capturing the AF ground state of the material.^49,50^ This value is obtained using a Hubbard parameter of *U* = 1.6 eV, which is selected since it reproduces the magnitude of *J*_int_ extracted from experimental findings and is in good agreement with the one obtained using HSE06 hybrid functionals (Figure S1).^50^ Therefore, we adopt this *U* = 1.6 eV for our subsequent analysis.

**Figure 1:**
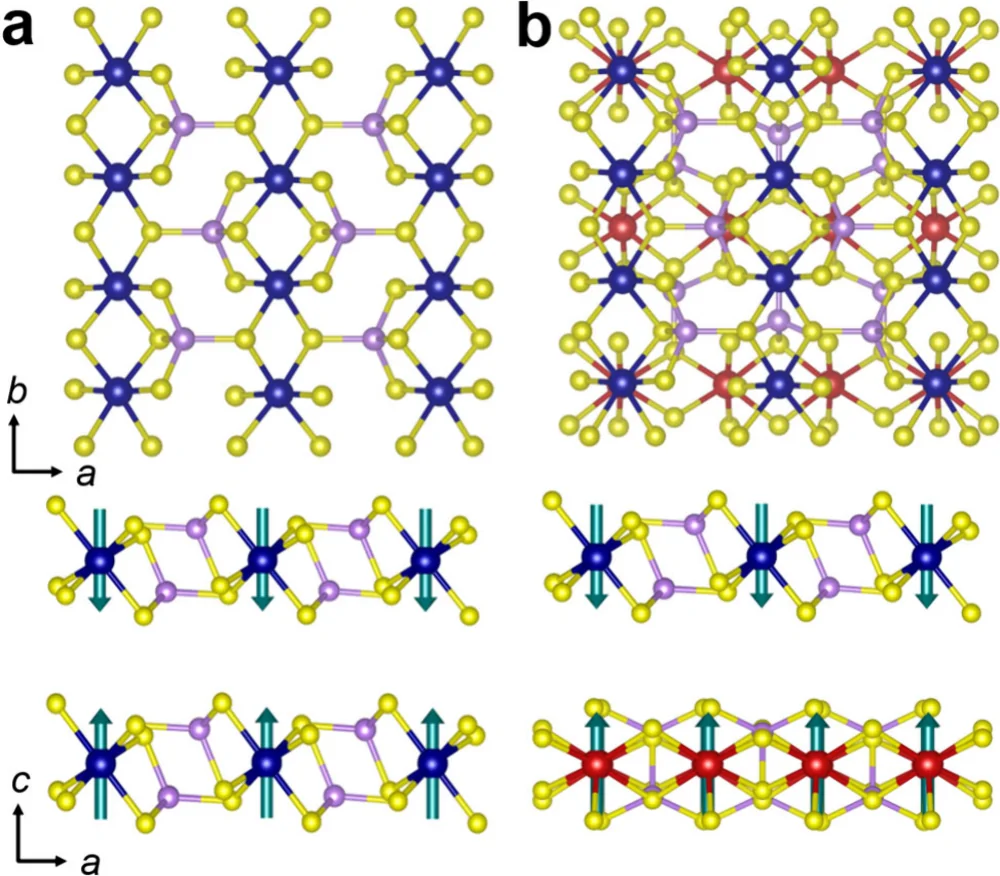
(a) Top and side views of pristine bilayer and (b) 90° twisted bilayer CrPS_4_. Color code: Cr (blue in the pristine bilayer; blue and red in the twisted bilayer for clarification), P (pink), and S (yellow). Green arrows indicate the spin direction.

The symmetry operations [*R*_*i*_||*R*_*j*_] for bilayer CrPS_4_ are defined as follows. The operation [*C*_2_|| P ] connects the two layers, where the transformation on the left (right) of the double vertical bar acts in spin (real crystallographic) space. Here, *C*_2_ corresponds to a spin inversion operation in spin space, while P denotes a spatial inversion. This symmetry relation imposes conventional AF order and leads to a spin-degenerate band structure (Figure S2).^45,49^ Furthermore, CrPS_4_ exhibits out-of-plane magnetic anisotropy arising from the combined effects of SOC and dipole–dipole interactions.^45^ Including both contributions, we obtain MAE_*bc*_ = *E*_*b*_ – *E*_*c*_ = 27.8 μeV/Cr and MAE_*ac*_ = *E*_*a*_ – *E*_*c*_ = 38.1 μeV/Cr, establishing the out-of-plane direction as the easy magnetization axis (Table S1).^44,45,49^

Then, we investigate a twisted bilayer configuration upon a rotation of 90°. To construct such a heterostructure, we create a 2 × 3 supercell with lattice parameters *a* = 21.76 Å and *b* = 21.84 Å. A small uniform strain (0.18%) is then applied to both layers to achieve commensurability, resulting in a square lattice with *a* = *b =* 21.80 Å (see Table S2 and Supporting Information Section 2.2 for a detailed discussion). In order to assess the stability of the selected stacking, we consider several configurations obtained by laterally displacing the rotated layer (Supporting Information Section 2.3). Notably, the structure shown in Figure 1b is the most stable among those analyzed, while all stackings retain a fully compensated net magnetization (Table S4).

The orthogonal twist breaks the [*C*_*2*_|| P ] symmetry. Instead, opposite spin sublattices are connected through [*C*_2_||*C*_*2(110)*_], where *C*_*2(110)*_ denotes a 180° spatial rotation around the diagonal in-plane direction. This, combined with the [*C*_2_ *||T*] symmetry (where *T* represents time-reversal symmetry), generates the conditions for altermagnetism.^29,32^ Upon rotation, the Brillouin zone of the twisted layer is correspondingly rotated (Figure 2). Specifically, the Γ–X direction along *k*_*x*_ transforms into Γ–X′ along *k*_*y*_, while Γ–Y rotates toward the *x* axis, which we denote as Γ–Y′. In the common supercell, the square lattice leads to two equivalent reciprocal lattice vectors lengths, denoted as Γ̅–X̅ and Γ̅–Y̅.

**Figure 2:**
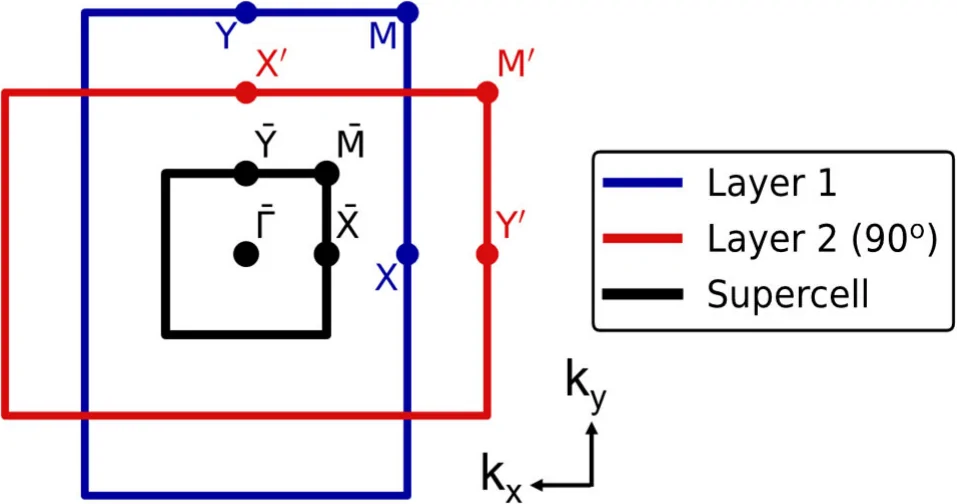
Schematic representation of the Brillouin zones of the untwisted layer (Layer 1), the twisted layer (Layer 2), and the common supercell employed for the 90° twisted CrPS_4_ heterostructure.

The calculated electronic band structure exhibits clear momentum-dependent spin splitting along Γ̅–X̅ and Γ̅–Y̅ (Figure 3a). For the rotated layer, the sequence of spin channels is inverted, reflecting the 90° rotation. The intrinsic band gap of the pristine bilayer is preserved upon twisting, showing a value of 1.2 eV. We also perform calculations for the original rectangular cell (*a* = 21.76 Å and *b* = 21.84 Å), in which strain is only applied to the rotated layer. Figure S3 shows that the bands remain essentially unchanged with respect to those of the square structure, confirming that the minimal applied strain does not affect the AM behavior. Furthermore, the additional stacking configurations show similar electronic band structures, retaining spin-split bands and largely preserving AM features (Figure S5).

**Figure 3:**
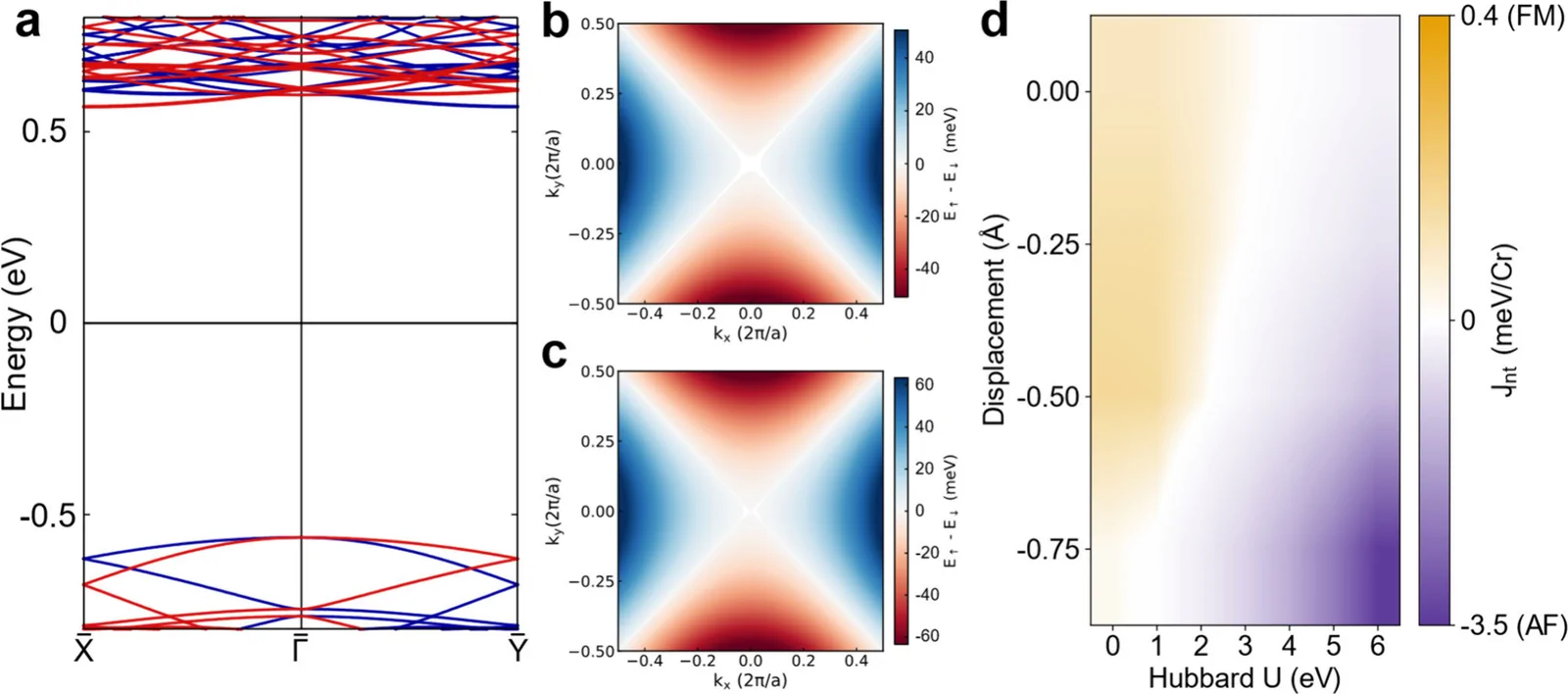
(a) Electronic band structure of twisted 90° bilayer CrPS_4_, together with the corresponding 2D maps of spin splitting for the (b) conduction band minimum and (c) valence band maximum. Blue and red color in the band structure denote spin up and spin down states, respectively. (d) Evolution of the interlayer exchange coupling *J*_int_ as a function of the on-site Hubbard *U* parameter and interlayer displacement. *J*_int_ is explicitly calculated for displacements of 0, −0.25, −0.50, and −0.75 Å and for *U* values from 0 to 6 eV in steps of 1 eV. Intermediate values in the color map are interpolated and used as a visual guide to highlight the overall trend. A displacement of 0.00 Å corresponds to the equilibrium interlayer distance of twisted bilayer CrPS_4_ (3.25 Å).

As depicted in Figure 3a, the spin splitting is larger in the valence band maximum than in the conduction band minimum. From the 2D spin splitting maps (Figures 3b,c), one can observe that the splitting vanishes at Γ̅ and increases continuously toward the Γ̅–X̅ and Γ̅–Y̅ directions, reaching a maximum value of ±68 meV at X̅ and Y̅, respectively. A similar trend is observed in the conduction band, with a maximum splitting of ±43 meV. In both cases, the momentum dependence of the splitting follows a characteristic *d*-wave symmetry, where its sign is reverted every 90°. It is also observed that a symmetry-protected nodal line emerges along the direction Γ̅–M̅. The inclusion of SOC effects leaves the band structure essentially unchanged (Figure S6), confirming the nonrelativistic origin of the splitting. Upon rotation, the preferential out-of-plane spin orientation is preserved, with an MAE of 22.3 μeV/Cr required to rotate spins toward the in-plane direction (Table S5).

Twisted bilayer CrPS_4_ exhibits an increased interlayer separation of 3.25 Å compared to 2.52 Å in the untwisted structure.^51^ This larger distance reduces the interlayer coupling, leading to a weak FM state with *J*_int_ = 0.085 meV/Cr. Owing to the vdW nature of CrPS_4_, this interaction can be effectively modified through uniaxial pressure applied on the out-of-plane direction. Indeed, experiments on bulk CrPS_4_ show that lattice compression decreases the interlayer spacing and enhances the AF character of *J*_int_ by 45%.^52^ A similar trend has been experimentally reported for CrSBr, where a ∼6% reduction of the interlayer distance enhances the AF coupling by 400%,^53^ reaching values of ∼1700% at higher pressure levels.^54^ Therefore, reducing the layer separation in twisted CrPS_4_ is expected to stabilize an AF ground state, providing a realistic route to experimentally access the AM phase.

To quantify this effect, we compute the evolution of *J*_int_ as a function of the interlayer distance for *U* = 1.6 eV (Figure 3d). Upon compression, *J*_int_ evolves continuously toward an AF regime, reaching −0.18 meV/Cr at a displacement of −0.75 Å, which corresponds to the interlayer separation of the pristine bilayer. Notably, this value is smaller compared to the untwisted case (*J*_int_ = −0.59 meV/Cr), despite the identical vertical spacing. This difference originates from their different stacking geometry. In the pristine structure, Cr atoms are vertically aligned between adjacent layers (Figure 1a), maximizing the AF coupling. On the other hand, the twisted configuration induces a reduced orbital overlap between interface atoms (Figure 1b), which weakens the AF interaction. While *J*_int_ is highly sensitive to interlayer variation, the magnetic anisotropy remains largely unaffected. Even under a maximum compression of −0.75 Å, the system preserves an out-of-plane anisotropy (Figure S7), thereby retaining altermagnetism.

Apart from the vertical displacement between adjacent layers, the magnetic properties of 2D materials can be significantly influenced by the effective on-site Coulomb interaction *U*, where its magnitude can be controlled via the dielectric environment or external fields.^55–57^ In particular, stronger environmental screening (e.g., high-dielectric substrates) reduces *U*, whereas weaker screening, such as low-dielectric substrates or suspended configurations, enhances it.^58,59^ Additionally, electrostatic gating can modify screening through gate-induced carriers, enabling a continuous renormalization of *U*, as demonstrated in CrBr_3_.^60^ Therefore, we explore the stabilization of the AM ground state upon variation of the Hubbard *U* parameter.

We find that increasing (decreasing) *U* leads to a continuous stabilization (reduction) of the AF interlayer coupling (Figure 3d). At the equilibrium interlayer distance (displacement = 0 Å), *J*_int_ evolves from a weak FM value of 0.085 meV/Cr at *U* = 1.6 eV to an enhanced AF character with *J*_int_ = −0.23 meV/Cr at *U* = 6 eV. This trend persists across all interlayer displacements, indicating that the combined effect of increasing *U* and layer compression progressively enhances the AF interaction, reaching *J*_int_ = −3.42 meV/Cr for *U* = 6 eV and a vertical displacement of −0.75 Å. Despite the substantial modification of *J*_int_, the out-of-plane spin orientation remains unchanged (Figure S7), while the maximum spin splitting decreases as one increases the Hubbard *U* and decreases the interlayer displacement (Figure S8).

In altermagnets, the anisotropic spin-momentum coupling leads to spin-polarized bands with direction-dependent group velocities, resulting in distinct conductivities for opposite spin channels. Consequently, the electrical transport becomes anisotropic and spin dependent. Figure 4a shows the electrical conductivity components σ_*xx*_, σ_*xy*_, and σ_*yy*_ resolved by a spin channel. A suppression of conductivity is observed around the Fermi level (*E*_f_), indicating a null carrier density regime consistent with a semiconducting gap, whereas away from *E*_f_ the longitudinal components σ_*xx*_ and σ_*yy*_ increase rapidly. The longitudinal conductivities exhibit a spin-dependent anisotropy governed by the 90° rotation, leading to σ_*xx*_^↑^ = σ_*yy*_^↓^ and vice versa. In contrast, the transverse conductivity σ_*xy*_ vanishes across the entire energy range due to the spin-degenerate bands along the nodal Γ̅–M̅ direction (Figures 3b and c).

**Figure 4:**
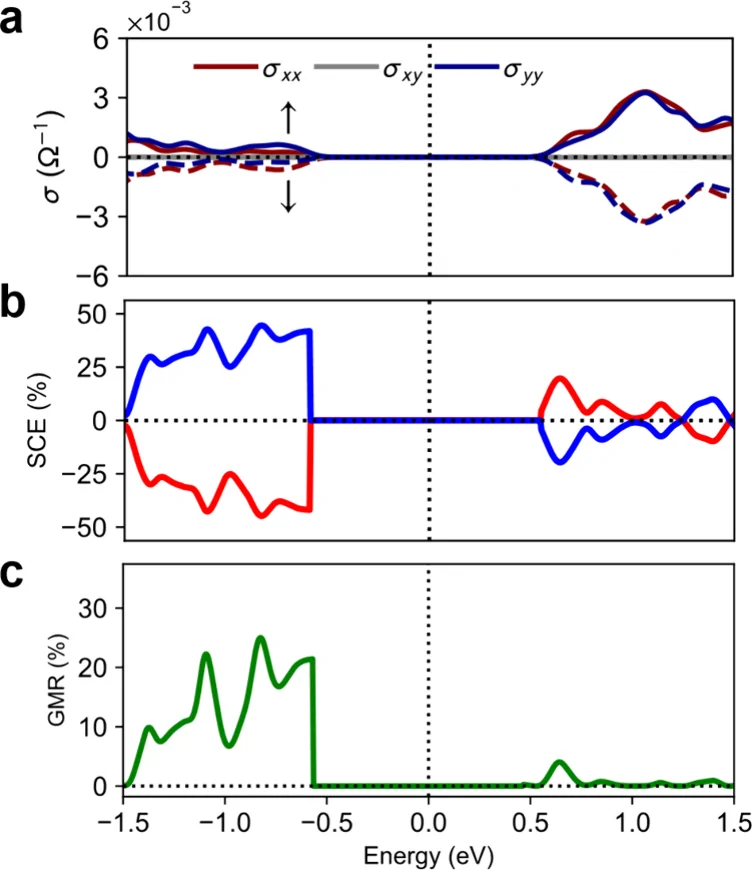
(a) Longitudinal (σ_*xx*_ and σ_*yy*_) and transverse (σ_*xy*_) conductivities. The solid (dashed) lines correspond to the spin up (down) contributions, with the latter plotted with negative sign to enable comparison. (b) SCE and (c) GMR for twisted CrPS_4_.

This symmetry-driven anisotropy has consequences for spin transport, as reflected from the calculation of the spin to charge efficiency (SCE), which is defined as

1 $$\mathrm{SCE}=\frac{\sigma_{ii}^{↑}-\sigma_{ii}^{↓}}{\sigma_{ii}^{↑}+\sigma_{ii}^{↓}}$$

Here, *ii* corresponds to either the *xx* or *yy* components, and SCE gives the ratio between the spin conductivity and charge conductivity.

Figure 4b shows that spin polarization exhibits opposite signs for the longitudinal *xx* and *yy* components, reaching magnitudes of up to 50% below *E*_f_. This indicates that charge currents flowing in orthogonal directions are carried by opposite spin channels. As a result, the system supports the generation of a spin current without requiring net magnetization, a hallmark of *d*-wave altermagnetism.^61^ Above *E*_f_, the SCE reaches 25%, comparable to values reported for RuO_2_ (27%)^62^ and V_2_Te_2_O (32%)^63^ and higher than the one for Mn_3_Sn (15%).^64^ Notably, magnetotransport measurements on CrPS_4_ indicate that the Fermi level is located close to the conduction band edge, typical of an n-type semiconductor.^49,65^ Therefore, slight electron doping can populate the conduction band edge and enable a substantial SCE.

Finally, Figure 4c shows the giant magnetoresistance (GMR), which is derived from the ratio of spin-resolved conductivities for both spin channels, *R*_σ_ = σ_*xx*_^↑^/σ_*xx*_^↓^ = σ_*xx*_^↑^/σ_*yy*_^↑^ = σ_*yy*_^↓^/σ_*xx*_^↓^. Specifically, the GMR has the form^66^

2 $$\mathrm{GMR}=\frac{1}{4}\left(R_{\sigma }+\frac{1}{R_{\sigma }}-2\right)$$

A significant GMR signal, reaching values up to 25%, is observed below the Fermi level, driven by the strong imbalance between σ_*xx*_^↑^ and σ_*xx*_^↓^. The GMR vanishes along the electronic gap, reflecting the null conductivity in this region, and remains comparatively small above the conduction band edge where the spin asymmetry is weaker. These results demonstrate that the interplay between symmetry-enforced anisotropy and the energy-dependent electronic structure enables both efficient spin current generation and sizable magnetoresistive effects.

In conclusion, we demonstrate that orthogonally twisted bilayer CrPS_4_ stands as a promising candidate for *d*-wave altermagnetism. Our results demonstrate that a 90° rotation breaks the [*C*_2_|| P ] symmetry and, consequently, opposite spin sublattices are connected through a [*C*_2_||*C*_*2(110)*_] operation, generating a sizable nonrelativistic spin splitting of up to 68 meV around the Fermi level. Our calculations show that the stability of the AM state can be reinforced by reducing the interlayer separation and through modification of the Coulomb screening. Additionally, the calculated band structure exhibits strong spin-dependent anisotropy, leading to a significant 50% (25%) spin to charge efficiency below (above) the Fermi level and sizable giant magnetoresistance. Overall, our findings identify twisted CrPS_4_ as an experimentally viable platform for altermagnetism and highlight twistronics as an effective route to engineer magnetism in 2D materials.

## Methods

Spin-polarized density functional theory (DFT) calculations for CrPS_4_ were performed using the Vienna Ab initio Simulation Package (VASP).^67^ The exchange–correlation energy was treated within the generalized gradient approximation (GGA). To construct the 90° twisted heterostructure, a 2 × 3 supercell is employed, with the corresponding atomic positions and lattice parameters provided in Table S2. To accurately describe the electronic and magnetic properties, the DFT+*U* approach was employed, with an effective Hubbard parameter of *U* = 1.6 eV. This value of *U* was selected as it reproduces the experimental reported *J*_int_ = −0.59 meV/Cr^50^ and yields results consistent with those obtained using the HSE06 hybrid functional (Figure S1). van der Waals (vdW) interactions between adjacent CrPS_4_ layers were treated using the DFT-D2 scheme. A comparison with the DFT-D3 method shows negligible differences, with both approaches reproducing *J*_int_ = −0.59 meV/Cr for *U* = 1.6 eV (Figure S2). Boltzmann transport calculations were performed using the Wannier90 code.^68^ The longitudinal and transversal conductivities were computed using a 120 × 120 × 1 crystal momentum mesh. The temperature was set to *T* = 300 K and the scattering rate to Γ = 10 meV. Additional calculations were performed for Γ = 1 meV and Γ = 100 meV (Figures S9–S11), showing that the conductivities are inversely proportional to Γ,^64^ while the SCE and GMR remain robust against variations of Γ.^61^

## Supplementary Material



## Notes

Bilayer CrPS_4_; orthogonally twisted bilayer CrPS_4_; atomic coordinates of 90° twisted bilayer CrPS_4_; construction of the 90° twisted supercell; effect of lateral stacking on altermagnetism; effect of anisotropy on the electronic properties and determination of MAE; effect of interlayer compression and Hubbard *U* on magnetic anisotropy and spin splitting; effect of scattering rate on conductivity, SCE, and GMR (PDF)
