# Supplementary material for: Emergent d‑Wave Altermagnetism in Orthogonally Twisted Bilayer CrPS4
Source: ACS Mater Au. 2026 Jun 17;6(5):1026–32. doi: 10.1021/acsmaterialsau.6c00086 (PMC13564144; doi:10.1021/acsmaterialsau.6c00086)
Supplement: Supplementary file 1 [file mg6c00086_si_001.pdf]

Supporting Information

Emergent *d*-wave altermagnetism in orthogonally twisted bilayer $CrPS_4$

Alberto M. Ruiz[a,‖], Diego López-Alcalá[a,‖], Rafael González-Hernández[b], José J. Baldoví[a,*]

[a]Instituto de Ciencia Molecular, Universitat de València, Catedrático José Beltrán 2, 46980 Paterna, Spain

[b]Departamento de Física y Geociencias, Universidad del Norte, Km. 5 Vía Antigua Puerto Colombia, 081007 Barranquilla, Colombia

[‖]These authors have contributed equally to this work

E-mail: j.jaime.baldovi@uv.es

Table of Contents

1. Bilayer $CrPS_4$ 2
2. Orthogonally twisted bilayer $CrPS_4$ 4
   2.1 Atomic coordinates of 90º twisted bilayer $CrPS_4$ 4
   2.2 Construction of the 90º twisted supercell 9
   2.3 Effect of lateral stacking on altermagnetism 10
   2.4 Effect of anisotropy on the electronic properties and determination of MAE 15
   2.5 Effect of interlayer compression and Hubbard U on magnetic anisotropy and spin splitting 16
   2.6 Effect of scattering rate on conductivity, SCE and GMR 17

1. Bilayer $CrPS_4$

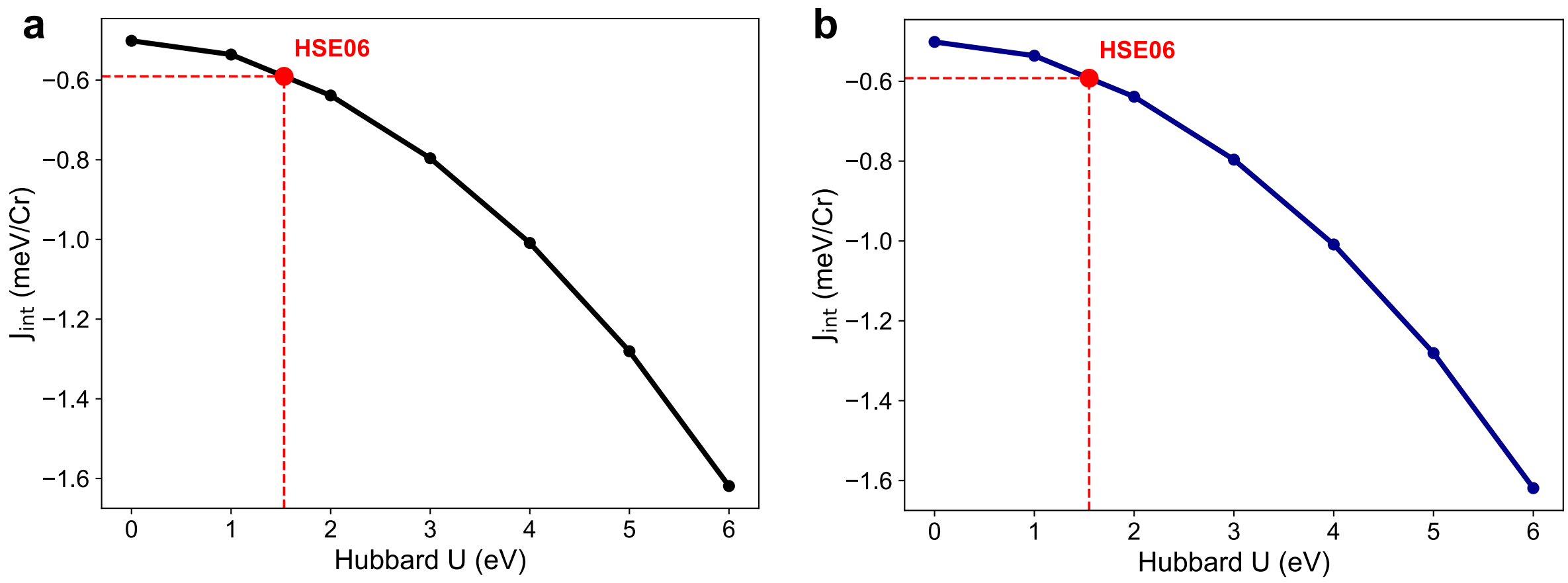

Figure S1. Evolution of J_{int} as a function of Hubbard U correction using the (a) GMD2 and (b) GMD3 vdW correction for bilayer $CrPS_4$. We mark the value of J_{int} obtained from Hybrid HSE06 functionals, which corresponds to a fitted U value of 1.6 eV in both cases.

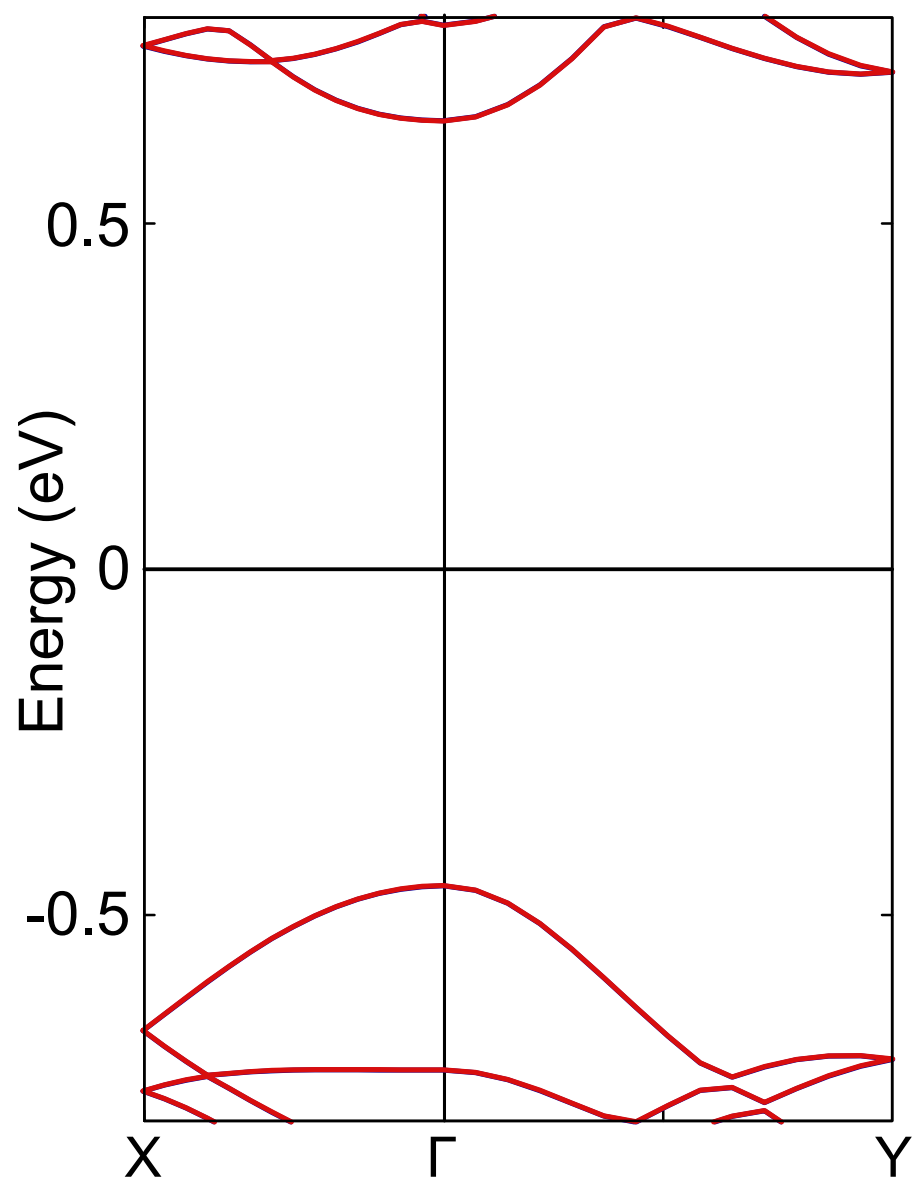

Figure S2. Electronic band structure of bilayer $CrPS_4$.

The magnetic anisotropy of $CrPS_4$ is evaluated by considering two contributions. The first arises from spin–orbit coupling (SOC) and is determined from the energy difference between in-plane and out-of-plane magnetization directions. The second contribution corresponds to the dipole-dipole anisotropy, which originates from dipolar interactions and is taken from previously reported calculations for $CrPS_4$.[1]

Table S1. SOC, dipole-dipole and total MAE for bilayer $CrPS_4$.

Contribution to MAE	MAE_{bc} (μeV/Cr)	MAE_{ac} (μeV/Cr)
SOC	45.9	40.6
Dipole-Dipole	-18.1	-2.5
Total	27.8	38.1

2. Orthogonally twisted bilayer $CrPS_4$

2.1 Atomic coordinates of 90° twisted bilayer $CrPS_4$

Table S2. Atomic coordinates and lattice parameters of the 90° twisted $CrPS_4$ bilayer structure.

```
Twisted CrPS4
   1.0000000000000000
    21.7899999999999991    0.0000000000000000    0.0000000000000000
     0.0000000000000000   21.7899999999999991    0.0000000000000000
     0.0000000000000000    0.0000000000000000   27.7434000000000012
   Cr   P    S
    48    48   192
Cartesian
   0.0001634250    7.2100131960    1.9163026540
   0.0001634250   14.4733465400    1.9119512480
  10.8951634250    7.2100131960    1.9135711530
  10.8951634250   14.4733465440    1.9053530610
  10.8951634250   -0.0533201300    1.9202492030
   0.0001634250   -0.0533201300    1.9132002330
   5.4475000000    7.2633333260    1.9061291810
   5.4475000000   14.5266666740    1.8787776940
  16.3425000000    7.2633333260    1.9078026110
  16.3425000000   14.5266666740    1.8735302510
  16.3425000000    0.0000000000    1.8838299250
   5.4475000000    0.0000000000    1.8867677990
   0.0000000000   10.8950000000    1.9188256840
   0.0000000000   18.1583333260    1.9167891280
  10.8950000000   10.8950000000    1.9095889680
  10.8950000000   18.1583333260    1.9222121700
  10.8950000000    3.6316666740    1.9015028850
   0.0000000000    3.6316666740    1.9111816220
   5.4476634250   10.8416798700    1.8999201260
   5.4476634250   18.1050131960    1.8927541080
  16.3426634250   10.8416798700    1.9029707900
  16.3426634250   18.1050131960    1.8901557800
  16.3426634250    3.5783465440    1.8759128540
   5.4476634250    3.5783465440    1.8755980360
   7.2100131960    0.0001634250    8.8391175410
  14.4733465440    0.0001634250    8.8428152680
   7.2100131960   10.8951634250    8.8420989480
  14.4733465440   10.8951634250    8.8495352270
  -0.0533201300   10.8951634250    8.8344992560
  -0.0533201300    0.0001634250    8.8417369520
  10.8416798700   16.3426634250    8.8533248820
  18.1050131960   16.3426634250    8.8632700340
  10.8416798700    5.4476634250    8.8566127540
  18.1050131960    5.4476634250    8.8614657810
   3.5783465440    5.4476634250    8.8786923750
   3.5783465440   16.3426634250    8.8776731730
  10.8950000000   21.7900000000    8.8363031050
  18.1583333260   21.7900000000    8.8376705630
  10.8950000000   10.8950000000    8.8458733410
  18.1583333260   10.8950000000    8.8323624810
   3.6316666740   10.8950000000    8.8536524550
   3.6316666740   21.7900000000    8.8435918080
   7.2633333260   16.3425000000    8.8478550950
  14.5266666740   16.3425000000    8.8797747670
   7.2633333260    5.4475000000    8.8497855740
  14.5266666740    5.4475000000    8.8755339440
   0.0000000000    5.4475000000    8.8676982330
   0.0000000000   16.3425000000    8.8699112310
   3.2237106550    9.0522560240    2.9568049430
   3.2237106550   16.3155893500    2.9352408040
  14.1187106550    9.0522560240    2.9615610390
  14.1187106550   16.3155893500    2.9284623610
```

14.1187106550	1.7889226760	2.9236776580
3.2237106550	1.7889226760	2.9257787090
8.6712106550	12.6839226760	2.9564475270
8.6712106550	19.9472560240	2.9772664680
19.5662106550	12.6839226760	2.9669959690
19.5662106550	19.9472560240	2.9698482490
19.5662106550	5.4205893500	2.9660690720
8.6712106550	5.4205893500	2.9600529470
7.6636519500	9.0522197000	0.8491021560
7.6636519500	16.3155530260	0.8328434270
18.5586519500	9.0522197000	0.8509255250
18.5586519500	16.3155530260	0.8298933470
18.5586519500	1.7888863740	0.8280574200
7.6636519500	1.7888863740	0.8281184370
2.2161519500	12.6838863740	0.8539306410
2.2161519500	19.9472197000	0.8548161910
13.1111519500	12.6838863740	0.8476737280
13.1111519500	19.9472197000	0.8611435710
13.1111519500	5.4205530260	0.8484239730
2.2161519500	5.4205530260	0.8545941220
9.0522197000	18.5586519500	9.9047789000
16.3155530260	18.5586519500	9.9231343270
9.0522197000	7.6636519500	9.9068184380
16.3155530260	7.6636519500	9.9212252990
1.7888863740	7.6636519500	9.9259515830
1.7888863740	18.5586519500	9.9253399650
12.6838863740	13.1111519500	9.9072753840
19.9472197000	13.1111519500	9.8933905510
12.6838863740	2.2161519500	9.9009018800
19.9472197000	2.2161519500	9.8996440990
5.4205530260	2.2161519500	9.9002883290
5.4205530260	13.1111519500	9.9067418010
9.0522560240	14.1186997600	7.7945175680
16.3155893500	14.1186997600	7.8248531030
9.0522560240	3.2236997600	7.7994920860
16.3155893500	3.2236997600	7.8188187010
1.7889226760	3.2236997600	7.8289279190
1.7889226760	14.1186997600	7.8302160990
12.6839226760	19.5661997600	7.7874380080
19.9472560240	19.5661997600	7.7843484850
12.6839226760	8.6711997600	7.7981491470
19.9472560240	8.6711997600	7.7769266240
5.4205893500	8.6711997600	7.7947656350
5.4205893500	19.5661997600	7.7884817870
1.4645059000	14.3867530840	0.0334248560
1.4645059000	21.6500864100	0.0344836830
12.3595059000	14.3867530840	0.0270307540
12.3595059000	21.6500864100	0.0413982230
12.3595059000	7.1234197360	0.0316134780
1.4645059000	7.1234197360	0.0359046490
3.9772306450	7.3500648040	3.7842327770
3.9772306450	14.6133981300	3.7334317310
14.8722306450	7.3500648040	3.7866973490
14.8722306450	14.6133981300	3.7231189460
14.8722306450	0.0867314560	3.7511450480
3.9772306450	0.0867314560	3.7561653340
6.9120059000	10.7550864100	0.0239825020
6.9120059000	18.0184197360	0.0122419550
17.8070059000	10.7550864100	0.0265020300
17.8070059000	18.0184197360	0.0090991910
17.8070059000	3.4917530840	0.0003785400
6.9120059000	3.4917530840	0.0000000000
1.4650833350	10.9806637460	0.0361854480
1.4650833350	18.2439970940	0.0371872740
12.3600833350	10.9806637460	0.0294177010
12.3600833350	18.2439970940	0.0426184710
12.3600833350	3.7173304200	0.0257259230

1.4650833350	3.7173304200	0.0341142250
6.9125833350	7.3489970940	0.0273987620
6.9125833350	14.6123304200	0.0044024800
17.8075833350	7.3489970940	0.0289714280
17.8075833350	14.6123304200	0.0004007560
17.8075833350	0.0856637460	0.0058944290
6.9125833350	0.0856637460	0.0070119960
9.7494561200	9.0523649740	0.7185921530
9.7494561200	16.3156983000	0.7152686200
20.6444561200	9.0523649740	0.7233794140
20.6444561200	16.3156983000	0.7151498010
20.6444561200	1.7890316260	0.7126600820
9.7494561200	1.7890316260	0.7112220320
4.3019561200	12.6840316260	0.7048730120
4.3019561200	19.9473649740	0.7057432140
15.1969561200	12.6840316260	0.7022542960
15.1969561200	19.9473649740	0.7054852330
15.1969561200	5.4206983000	0.7055116310
4.3019561200	5.4206983000	0.7063903090
9.4867123000	12.6843076400	1.0295254580
9.4867123000	19.9476409660	1.0496806650
20.3817123000	12.6843076400	1.0395274750
20.3817123000	19.9476409660	1.0423524100
20.3817123000	5.4209743140	1.0389557380
9.4867123000	5.4209743140	1.0327575600
4.0392123000	9.0526409660	1.0296548960
4.0392123000	16.3159743140	1.0082135410
14.9342123000	9.0526409660	1.0339758710
14.9342123000	16.3159743140	1.0021527850
14.9342123000	1.7893076400	0.9972114030
4.0392123000	1.7893076400	0.9989046880
9.4259617800	14.3852495740	3.7750647530
9.4259617800	21.6485829000	3.8012542020
20.3209617800	14.3852495740	3.7859713360
20.3209617800	21.6485829000	3.7863813400
20.3209617800	7.1219162260	3.7933690050
9.4259617800	7.1219162260	3.7939142700
3.9784617800	10.7535829000	3.7664508450
3.9784617800	18.0169162260	3.7676184210
14.8734617800	10.7535829000	3.7742185440
14.8734617800	18.0169162260	3.7645635370
14.8734617800	3.4902495740	3.7317698380
3.9784617800	3.4902495740	3.7305776840
9.4247306450	10.9817314560	3.7845962990
9.4247306450	18.2450648040	3.8035233060
20.3197306450	10.9817314560	3.7984513840
20.3197306450	18.2450648040	3.7974582440
20.3197306450	3.7183981300	3.7839125650
9.4247306450	3.7183981300	3.7648329720
1.1383531800	9.0525465500	3.1072568360
1.1383531800	16.3158798760	3.0995020480
12.0333531800	9.0525465500	3.1029500780
12.0333531800	16.3158798760	3.0986910670
12.0333531800	1.7892132240	3.0942574250
1.1383531800	1.7892132240	3.0961474610
6.5858531800	12.6842132240	3.0907680240
6.5858531800	19.9475465500	3.0895050450
17.4808531800	12.6842132240	3.0927302410
17.4808531800	19.9475465500	3.0872083830
17.4808531800	5.4208798760	3.0937505020
6.5858531800	5.4208798760	3.0925674930
1.4085818650	12.6840824840	2.7862689820
1.4085818650	19.9474158100	2.7870485610
12.3035818650	12.6840824840	2.7795820030
12.3035818650	19.9474158100	2.7938657600
12.3035818650	5.4207491360	2.7800115360
1.4085818650	5.4207491360	2.7864470230

6.8560818650	9.0524158100	2.7800835130
6.8560818650	16.3157491360	2.7630022020
17.7510818650	9.0524158100	2.7821553680
17.7510818650	16.3157491360	2.7596972220
17.7510818650	1.7890824840	2.7577575840
6.8560818650	1.7890824840	2.7580741110
14.3867530840	12.3595059000	10.7279338900
21.6500864100	12.3595059000	10.7134173200
14.3867530840	1.4645059000	10.7215254000
21.6500864100	1.4645059000	10.7203557900
7.1234197360	1.4645059000	10.7193230200
7.1234197360	12.3595059000	10.7239056000
10.7550864100	17.8070059000	10.7295820400
18.0184197360	17.8070059000	10.7440528700
10.7550864100	6.9120059000	10.7322605400
18.0184197360	6.9120059000	10.7418501700
3.4917530840	6.9120059000	10.7540910500
3.4917530840	17.8070059000	10.7529957700
10.9806637460	12.3600833350	10.7258852700
18.2439970940	12.3600833350	10.7121329800
10.9806637460	1.4650833350	10.7188232300
18.2439970940	1.4650833350	10.7172786000
3.7173304200	1.4650833350	10.7207359400
3.7173304200	12.3600833350	10.7294906200
7.3489970940	17.8075833350	10.7267955300
14.6123304200	17.8075833350	10.7525416000
7.3489970940	6.9125833350	10.7285661500
14.6123304200	6.9125833350	10.7496877000
0.0856637460	6.9125833350	10.7471065600
0.0856637460	17.8075833350	10.7475906400
9.0523649740	20.6444452250	10.0315894100
16.3156983000	20.6444452250	10.0386241600
9.0523649740	9.7494452250	10.0367326900
16.3156983000	9.7494452250	10.0388896800
1.7890316260	9.7494452250	10.0432452400
1.7890316260	20.6444452250	10.0414628000
12.6840316260	15.1969452250	10.0524704000
19.9473649740	15.1969452250	10.0481948900
12.6840316260	4.3019452250	10.0501321100
19.9473649740	4.3019452250	10.0485447300
5.4206983000	4.3019452250	10.0483633400
5.4206983000	15.1969452250	10.0490981400
12.6843076400	20.3817123000	9.7151882680
19.9476409660	20.3817123000	9.7121092870
12.6843076400	9.4867123000	9.7253488790
19.9476409660	9.4867123000	9.7049123860
5.4209743140	9.4867123000	9.7222917120
5.4209743140	20.3817123000	9.7157939480
9.0526409660	14.9342123000	9.7222087190
16.3159743140	14.9342123000	9.7512252570
9.0526409660	4.0392123000	9.7267526210
16.3159743140	4.0392123000	9.7459510020
1.7893076400	4.0392123000	9.7557534260
1.7893076400	14.9342123000	9.7566253660
14.3852495740	20.3209508850	6.9682297710
21.6485829000	20.3209508850	6.9683111830
14.3852495740	9.4259508850	6.9793597860
21.6485829000	9.4259508850	6.9530585390
7.1219162260	9.4259508850	6.9613884930
7.1219162260	20.3209508850	6.9618135000
10.7535829000	14.8734508850	6.9828305150
18.0169162260	14.8734508850	6.9888264590
10.7535829000	3.9784508850	6.9907012760
18.0169162260	3.9784508850	6.9865247910
3.4902495740	3.9784508850	7.0236938360
3.4902495740	14.8734508850	7.0216690250
10.9817314560	20.3197197500	6.9564200620

18.2450648040	20.3197197500	6.9567454570
10.9817314560	9.4247197500	6.9704696440
18.2450648040	9.4247197500	6.9506611680
3.7183981300	9.4247197500	6.9901910520
3.7183981300	20.3197197500	6.9704642440
7.3500648040	14.8722197500	6.9689458410
14.6133981300	14.8722197500	7.0301929030
7.3500648040	3.9772197500	6.9717094530
14.6133981300	3.9772197500	7.0209900950
0.0867314560	3.9772197500	6.9990543810
0.0867314560	14.8722197500	7.0031548150
9.0525465500	12.0333422850	7.6522882620
16.3158798760	12.0333422850	7.6551350630
9.0525465500	1.1383422850	7.6479052680
16.3158798760	1.1383422850	7.6544006830
1.7892132240	1.1383422850	7.6581633520
1.7892132240	12.0333422850	7.6598634930
12.6842132240	17.4808422850	7.6614354210
19.9475465500	17.4808422850	7.6657747390
12.6842132240	6.5858422850	7.6638593190
19.9475465500	6.5858422850	7.6639413990
5.4208798760	6.5858422850	7.6619412300
5.4208798760	17.4808422850	7.6604282750
12.6840824840	12.3035818650	7.9757178070
19.9474158100	12.3035818650	7.9608075090
12.6840824840	1.4085818650	7.9687029700
19.9474158100	1.4085818650	7.9676116950
5.4207491360	1.4085818650	7.9686405770
5.4207491360	12.3035818650	7.9754770410
9.0524158100	17.7510818650	7.9738769870
16.3157491360	17.7510818650	7.9934847160
9.0524158100	6.8560818650	7.9761770570
16.3157491360	6.8560818650	7.9912428070
1.7890824840	6.8560818650	7.9961304330
1.7890824840	17.7510818650	7.9957861540

2.2 Construction of the 90º twisted supercell

In the 2×3 supercell, the rectangular $CrPS_4$ supercell has nearly identical but not exactly equal in-plane dimensions, a = 21.76 Å and b = 21.84 Å. When the second layer is rotated by 90°, its crystallographic axes are interchanged with respect to the reference layer.

In particular, original b direction of Layer 2 becomes aligned with the a direction of Layer 1, while the original a direction of Layer 2 becomes aligned with the b direction of Layer 1. Therefore, if one kept the lattice parameters fixed to those of Layer 1, namely a = 21.76 Å and b = 21.84 Å, the rotated Layer 2 would be forced to adopt a' = 21.76 Å along a direction that naturally corresponds to 21.84 Å, and b' = 21.84 Å along a direction that naturally corresponds to 21.76 Å. This would introduce a larger strain acting solely on the rotated Layer 2.

To avoid this asymmetric strain distribution, we use the symmetric intermediate square cell a = b = 21.80 Å. This configuration distributes the very small mismatch equally between both layers and provides the closest periodic approximation to the expected 90° twisted bilayer under experimental conditions. Indeed, this configuration of a = b = 21.80 Å is more stable than the one with a = 21.76 Å and b = 21.84 Å (Table S3).

Table S3. Relative total energy of 90° twisted bilayer $CrPS_4$ calculated for the symmetric square cell used in the main text and for the rectangular cell. Energies are given with respect to the a = b = 21.80 Å configuration.

Lattice Parameters	ΔE(meV)
a = b = 21.80 Å	0.00
a = 21.76 Å, b = 21.84 Å	21.53

Nevertheless, the computed the electronic band structure for twisted $CrPS_4$ with a = 21.76 Å and b = 21.84 Å (Figure S3) shows that the altermagnetic features remain intact with respect those with a = b = 21.80 Å considered in the main text (Figure 3a).

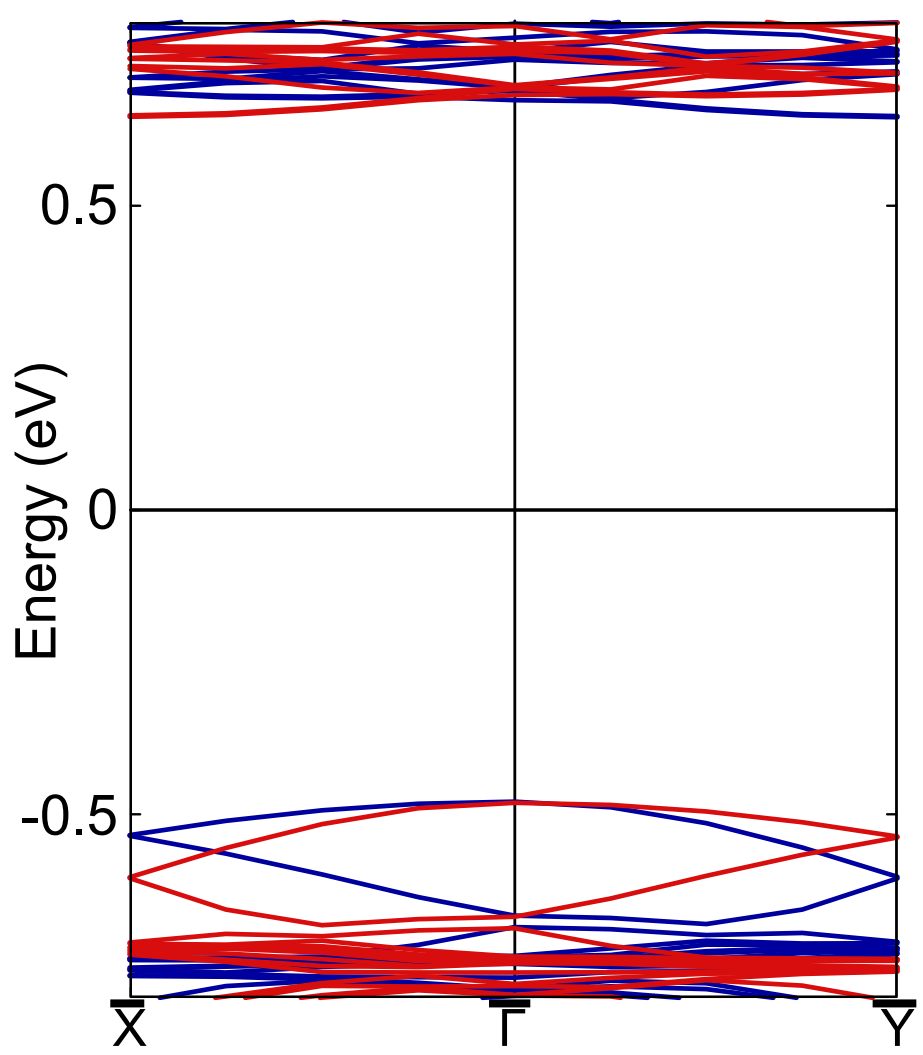

Figure S3. Electronic band structure of 90º twisted $CrPS_4$ with lattice parameters a = 21.76 Å and b = 21.84 Å.

2.3Effect of lateral stacking on altermagnetism

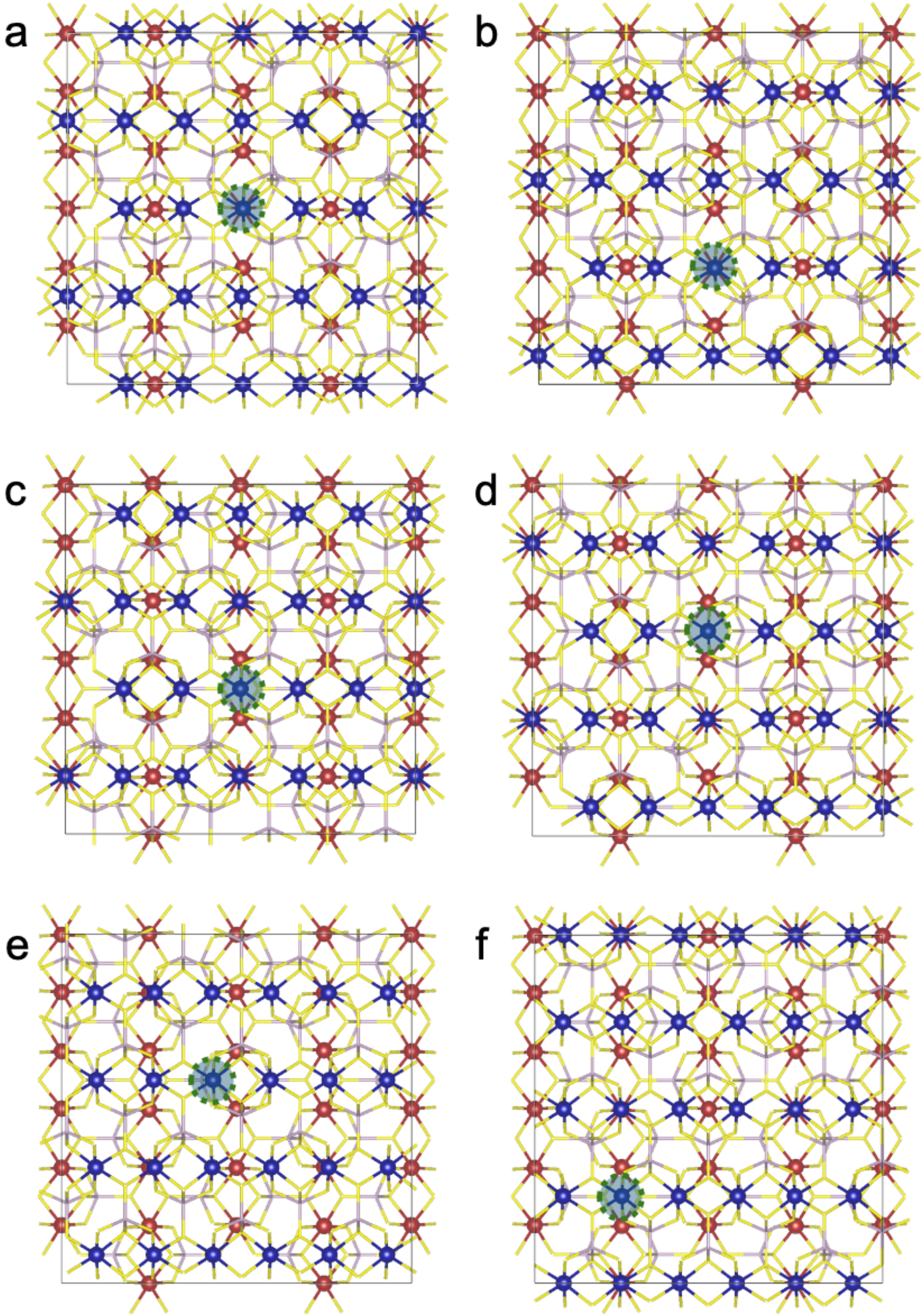

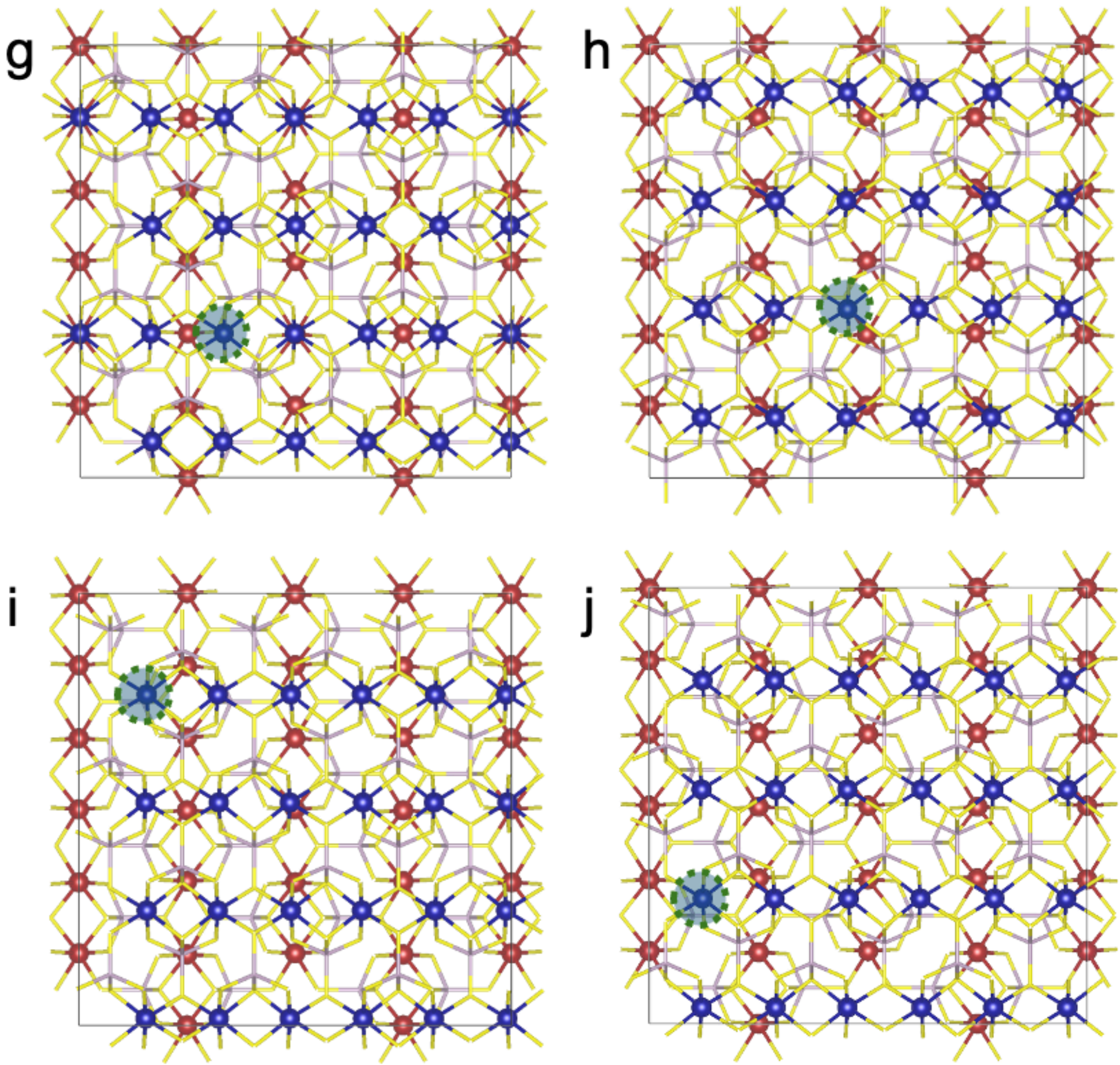

Figure S4. Top views of the 90° twisted bilayer $CrPS_4$ stacking configurations. (a) Twisted $CrPS_4$ structure used in the main text, and (b–j) laterally displaced configurations 1–9. For simplicity, only Cr atoms are shown. Cr atoms belonging to the top and bottom layers are shown in blue and red, respectively. The circled Cr atom is used as a visual reference to highlight the relative lateral displacement between the two layers.

Table S4. Relative total energy and total magnetic moment of the 90° twisted bilayer $CrPS_4$ stacking configurations considered in the lateral-displacement analysis. Energies are given with respect to the stacking configuration used in the main text. All configurations retain a fully compensated antiferromagnetic state with null net magnetization.

Configuration	ΔE(meV)	M_{tot} (μ_B/unit cell)
Structure used in this work	0.00	0.00
1	50.88	0.00
2	102.98	0.00
3	99.25	0.00
4	171.52	0.00
5	91.75	0.00
6	96.48	0.00
7	165.89	0.00
8	75.87	0.00
9	125.07	0.00

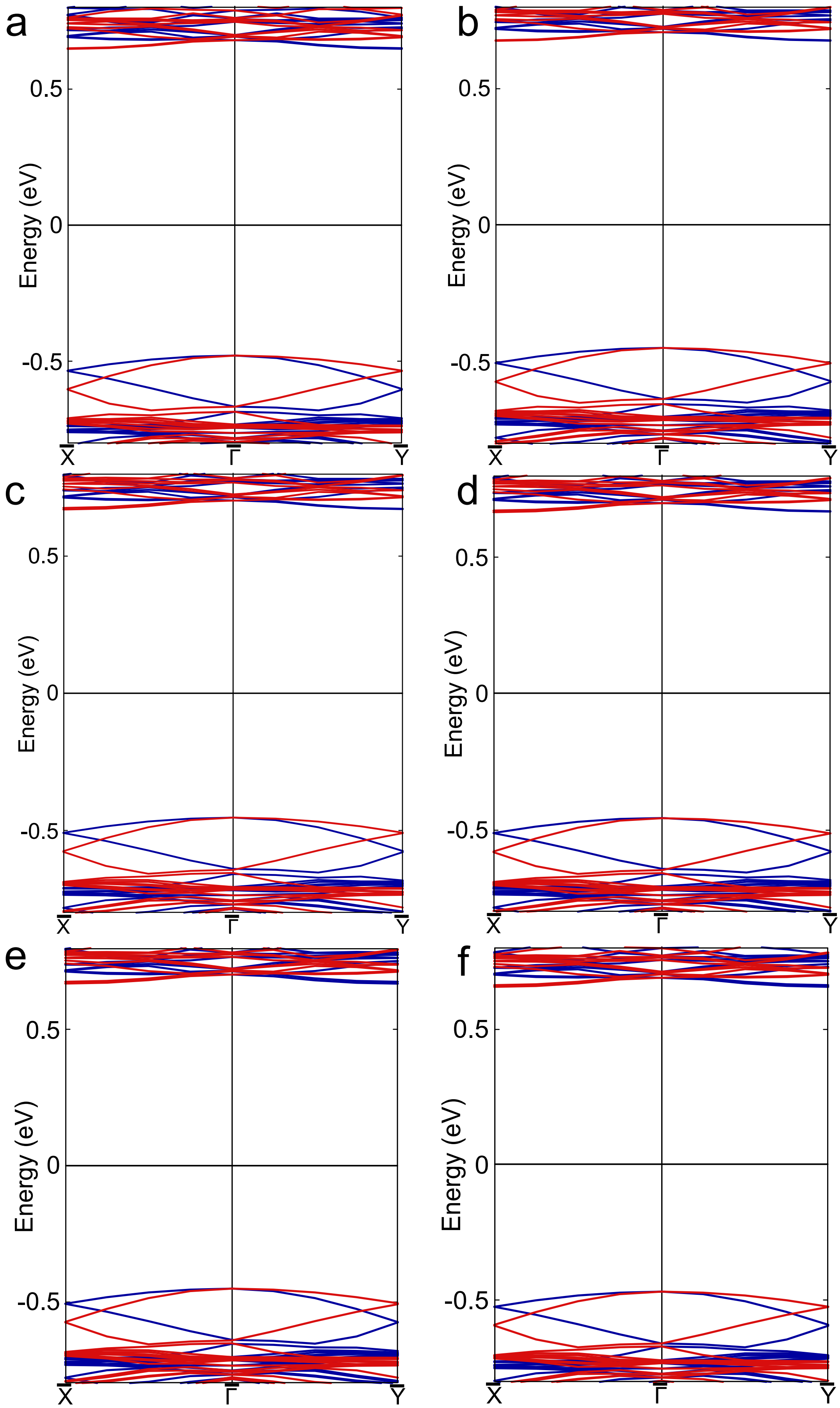

a
0.5
Energy (eV) 0
-0.5
X̄
Γ̄
Ȳ
b
0.5
Energy (eV) 0
-0.5
X̄
Γ̄
Ȳ
c
0.5
Energy (eV) 0
-0.5
X̄
Γ̄
Ȳ
d
0.5
Energy (eV) 0
-0.5
X̄
Γ̄
Ȳ
e
0.5
Energy (eV) 0
-0.5
X̄
Γ̄
Ȳ
f
0.5
Energy (eV) 0
-0.5
X̄
Γ̄
Ȳ

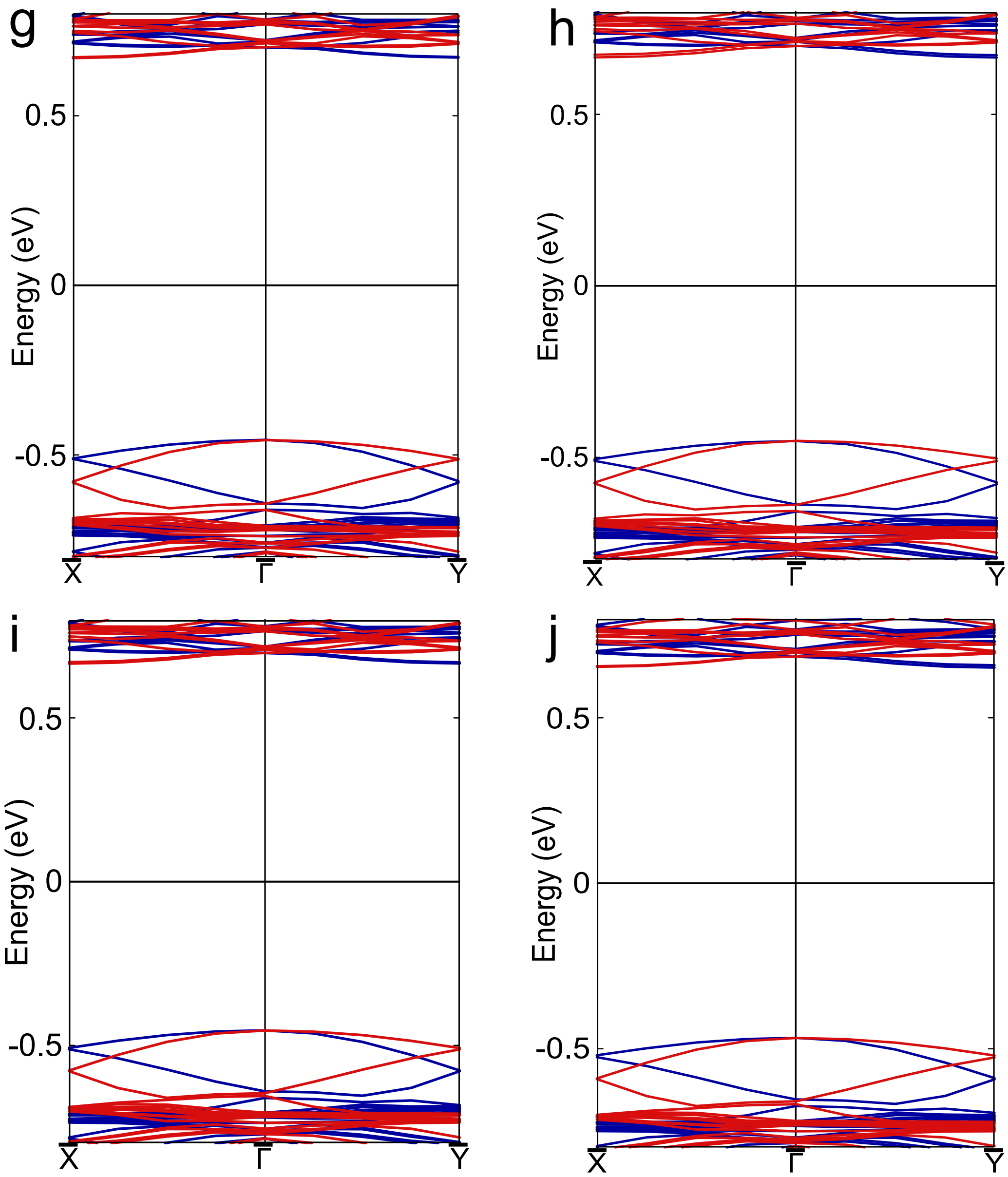

Figure S5. Electronic band structure of twisted $CrPS_4$ in the (a) configuration used in the main text, and (b–j) laterally displaced configurations 1–9. Blue and red color in the band structure denotes spin up and spin down states, respectively.

2.4 Effect of anisotropy on the electronic properties and determination of MAE

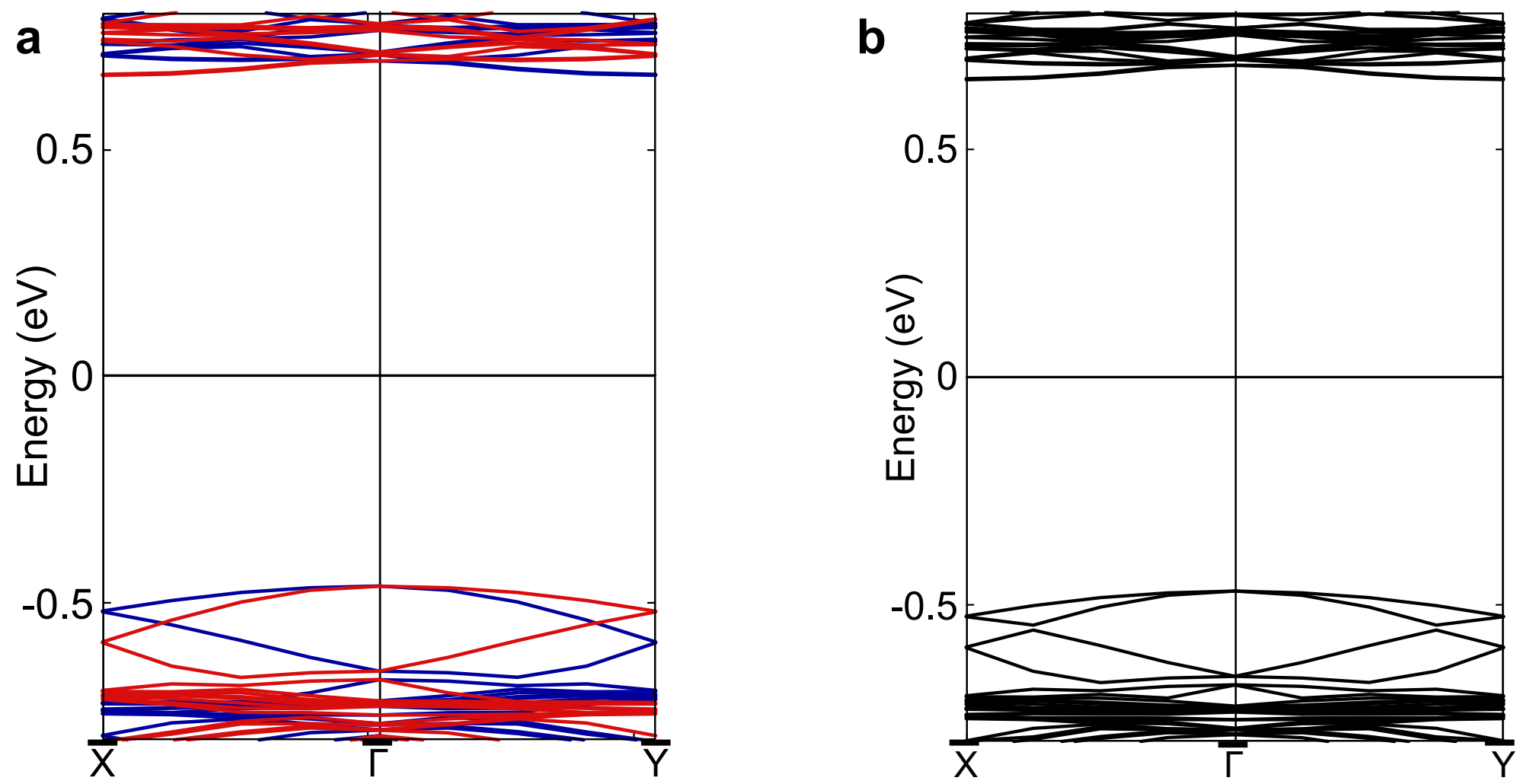

Figure S6. Electronic band structure of 90º twisted $CrPS_4$ calculated (a) without SOC and (b) with SOC.

For the calculation of the MAE in the 90° twisted configuration of $CrPS_4$, the SOC contribution is obtained from the energy difference between in-plane and out-of-plane magnetization orientations. On the other hand, the dipole–dipole contribution is assumed to be the same as in the pristine bilayer $CrPS_4$. In this case, the dipolar term favours an in-plane magnetic configuration along the *b* crystallographic directions for each $CrPS_4$ layer. Considering both terms, spins align along the out-of-plane directions, showing a value of MAE = 22.3 μeV/Cr.

Table S5. SOC, dipole-dipole and total MAE for 90º twisted $CrPS_4$.

Contribution to MAE	MAE = $E_{ip} - E_{oop}$ (μeV/Cr)
SOC	40.4
Dipole-Dipole	-18.1
Total	22.3

2.5 Effect of interlayer compression and Hubbard U on magnetic anisotropy and spin splitting

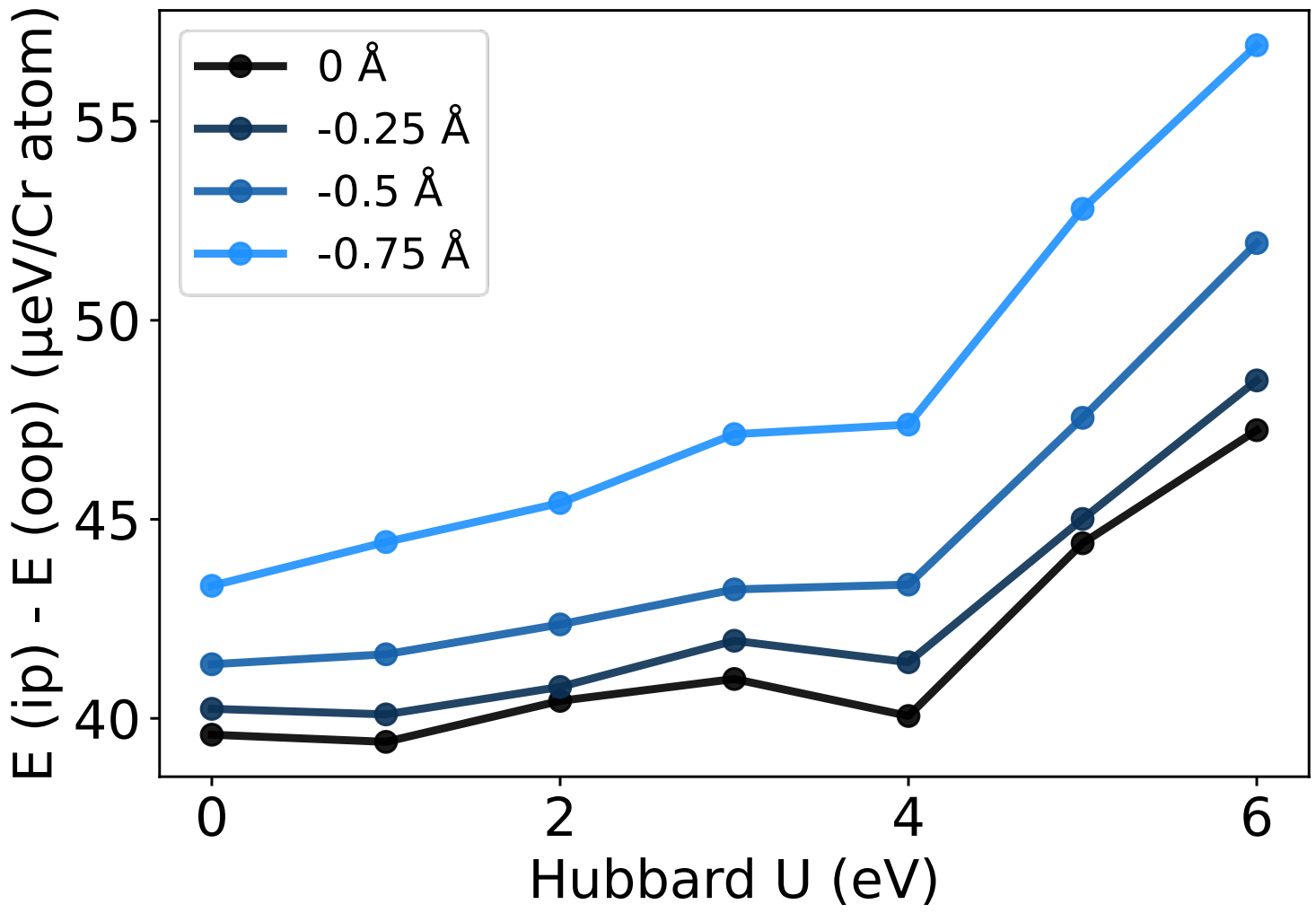

Figure S7. Evolution of SOC MAE obtained from the energy difference between spins pointing along the in-plane direction $E_{(ip)}$ and out-of-plane direction $E_{(oop)}$ for twisted $CrPS_4$ as a function of interlayer spacing and Hubbard U.

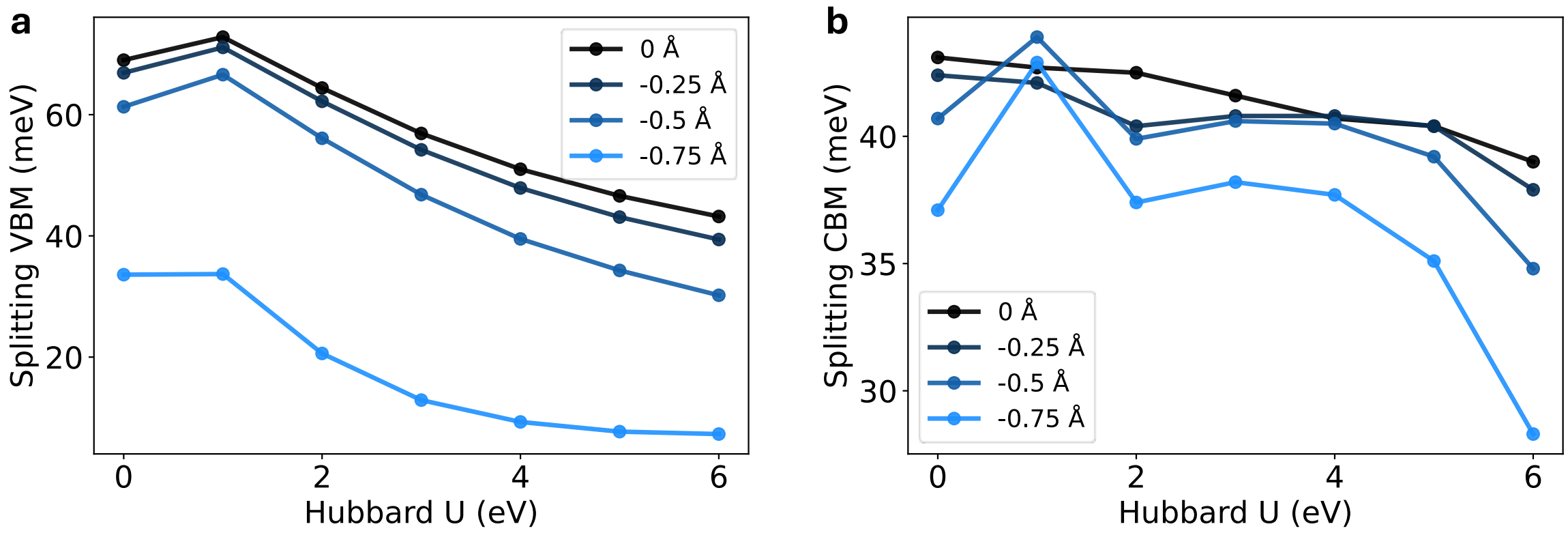

Figure S8. Evolution of maximum spin splitting as a function of Hubbard U correction in (a) valence band maximum (VBM) and (b) conduction band minimum (CBM).

2.6 Effect of scattering rate on conductivity, SCE and GMR

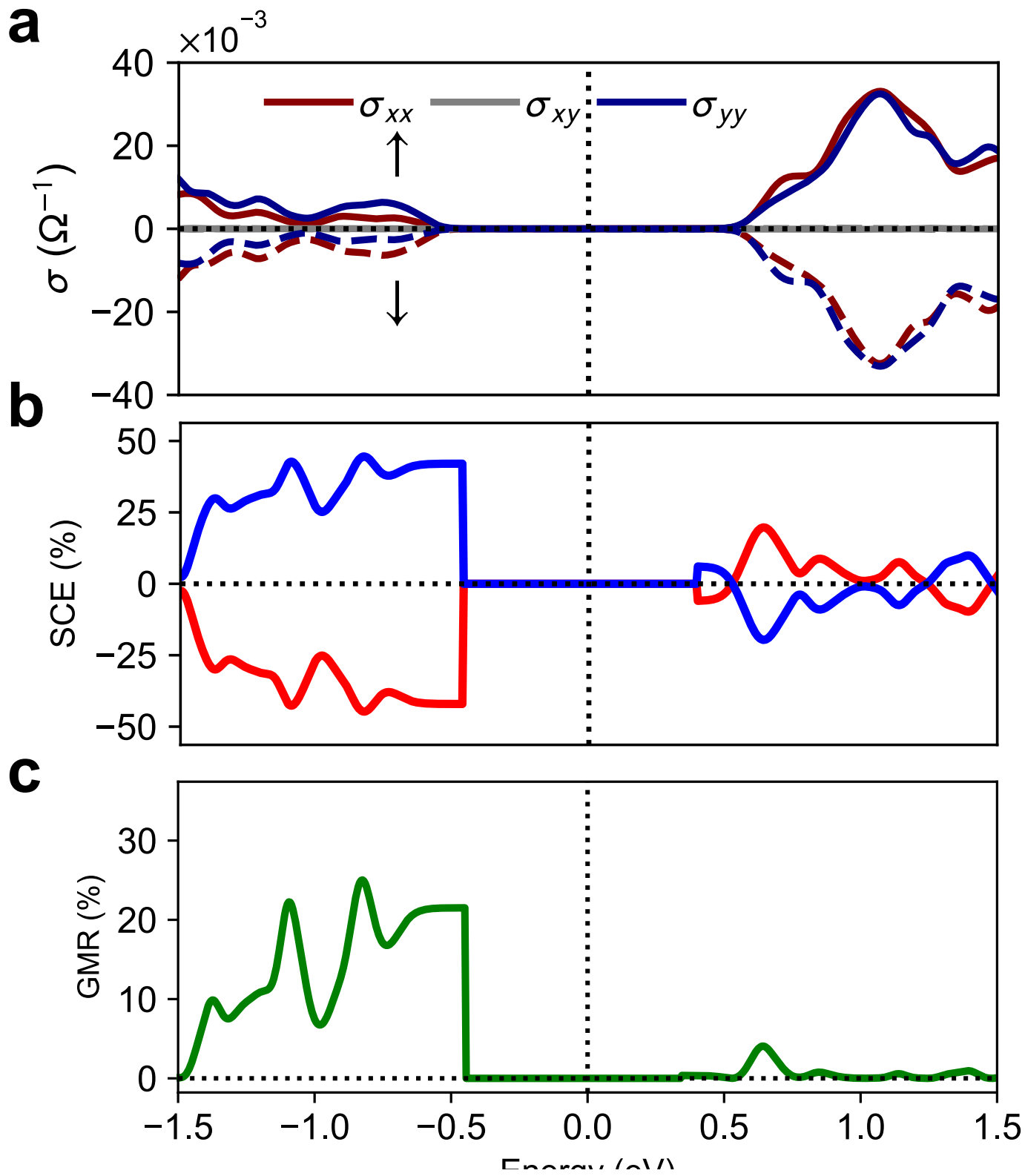

Figure S9. Conductivities, SCE and GMR for 90º twisted $CrPS_4$. (a) Longitudinal (σ_{xx} and σ_{yy}) and transverse (σ_{xy}) conductivities. The solid (dashed) lines correspond to the spin up (down) contributions, with the latter plotted with negative sign to enable comparison. (b) Corresponding SCE and (c) GMR, calculated using Equations 1 and 2 from the main text. The properties are computed using scattering rate (Γ) = 1 meV.

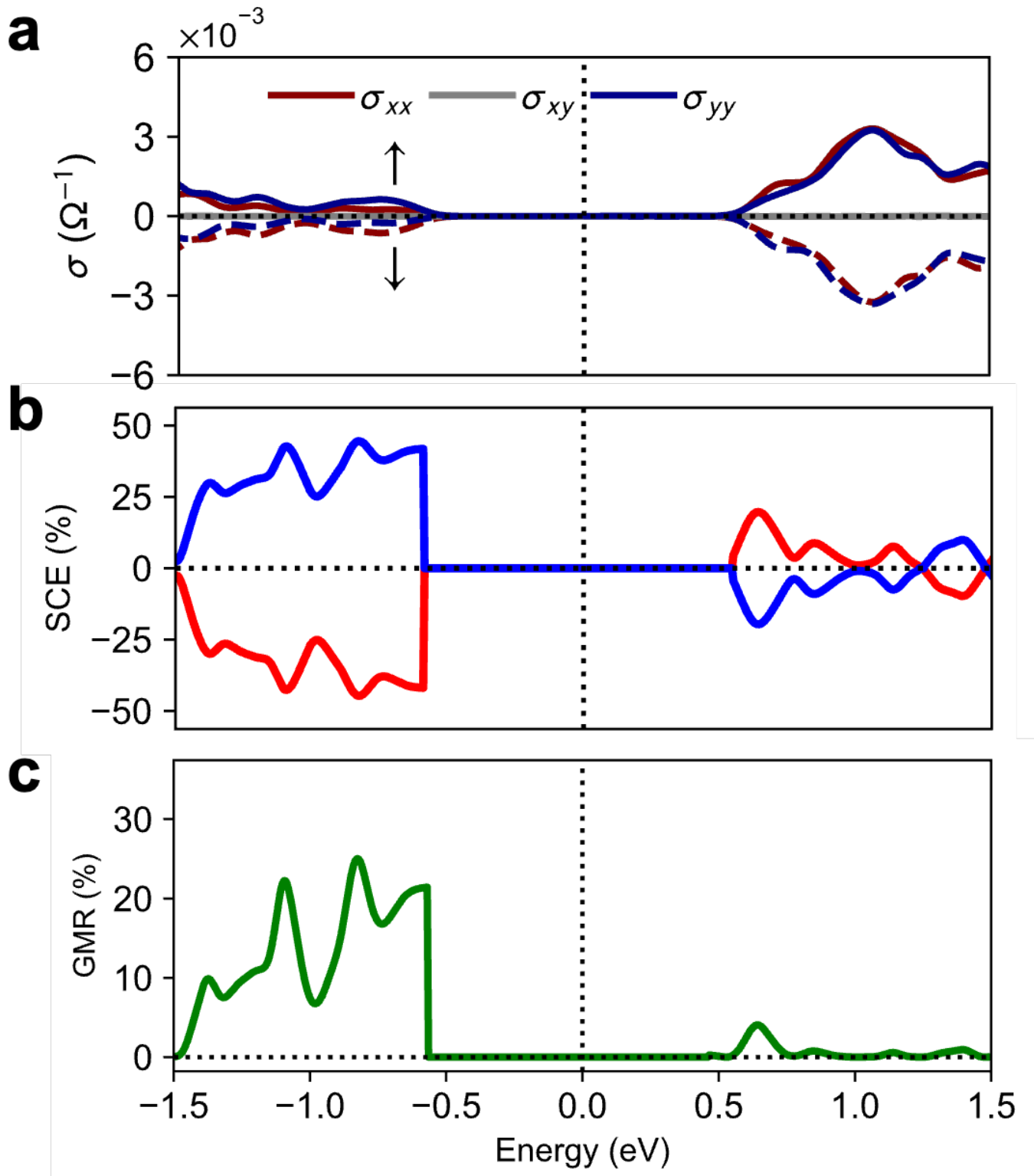

Figure S10. Same as Figure S9, but with Γ = 10 meV.

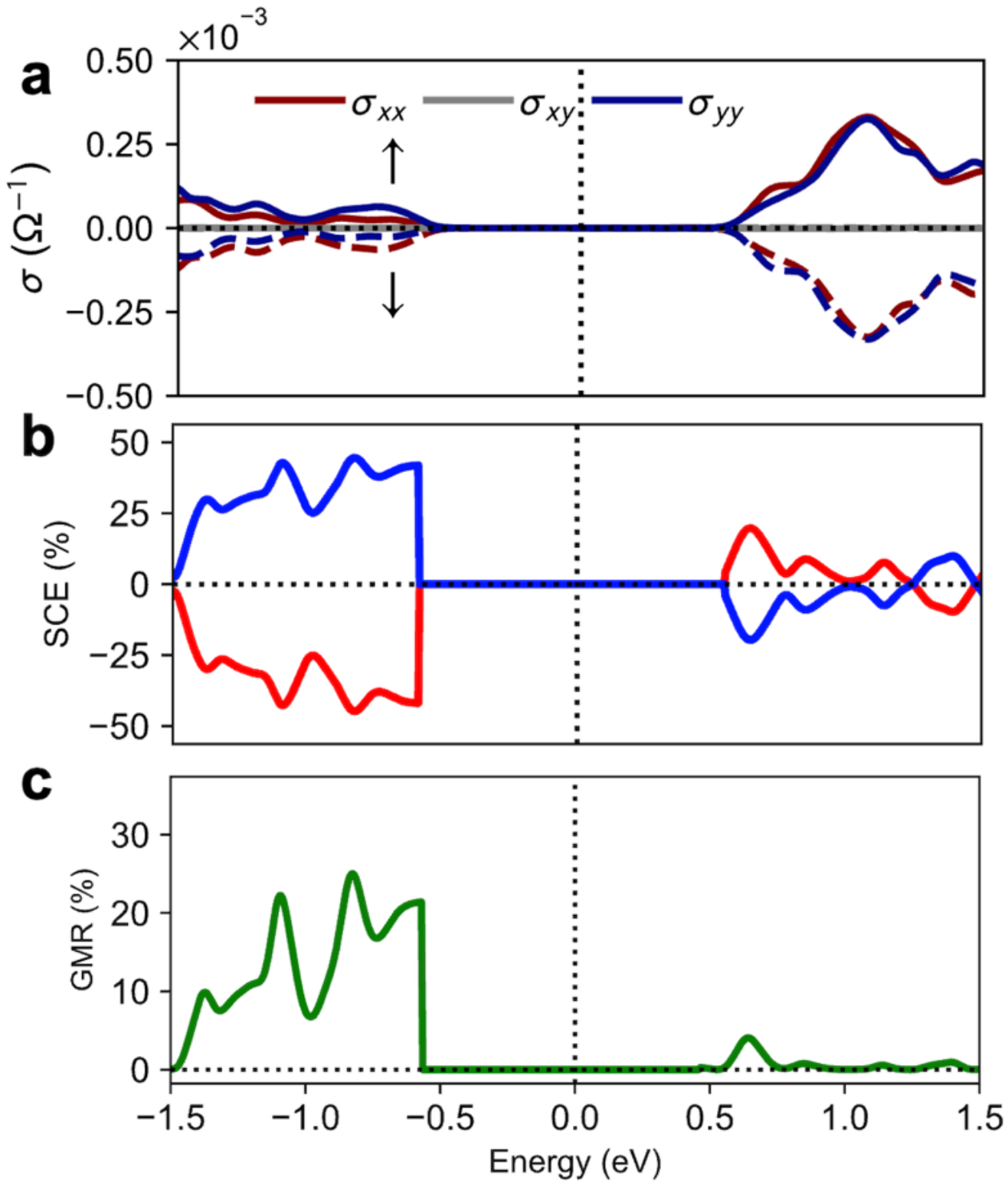

Figure S11. Same as Figure S9, but with Γ = 100 meV.

Figures S9-11 show the calculations of conductivities, SCE and GMR using Γ = 1, 10 and 100 meV, respectively. From our results we can extract that the conductivities are inversely proportional to the scattering rate Γ, while the CSE and GMR are robust against variations of Γ.[2–4]

REFERENCES

(1) Ruiz, A. M.; López-Alcalá, D.; Rivero-Carracedo, G.; Shumilin, A.; Baldoví, J. J. Controlling Magnetism in the 2D van Der Waals Antiferromagnet $CrPS_4$ via Ion Intercalation. *Nano Lett.* **2026**, *26* (9), 3018–3025.

(2) Železný, J.; Zhang, Y.; Felser, C.; Yan, B. Spin-Polarized Current in Noncollinear Antiferromagnets. *Phys. Rev. Lett.* **2017**, *119* (18), 187204.

(3) González-Hernández, R.; Šmejkal, L.; Výborný, K.; Yahagi, Y.; Sinova, J.; Jungwirth, T.; Železný, J. Efficient Electrical Spin Splitter Based on Nonrelativistic Collinear Antiferromagnetism. *Phys. Rev. Lett.* **2021**, *126* (12), 127701.

(4) Lai, J.; Yu, T.; Liu, P.; Liu, L.; Xing, G.; Chen, X.-Q.; Sun, Y. *d*-Wave Flat Fermi Surface in Altermagnets Enables Maximum Charge-to-Spin Conversion. *Phys. Rev. Lett.* **2025**, *135* (25), 256702.
